# Challenges for Diagnostic Clarity for Post-stroke Cognitive Impairment and Behavioural Issues in Middle-Income Countries: Case Studies From Malaysia

**DOI:** 10.3389/fneur.2021.628876

**Published:** 2021-06-02

**Authors:** Kwong Hsia Yap, Narelle Warren, Pascale Allotey, Daniel Reidpath

**Affiliations:** ^1^Jeffrey Cheah School of Medicine and Health Sciences, Monash University Malaysia, Subang Jaya, Malaysia; ^2^School of Social Sciences, Monash University, Clayton, VIC, Australia; ^3^International Institute for Global Health, United Nations University, Kuala Lumpur, Malaysia; ^4^Health Systems and Population Studies Division, icddr, b, Dhaka, Bangladesh

**Keywords:** post-stroke, cognitive impairment, cognitive screening, under-diagnosis, Malaysia

## Abstract

Following stroke, individuals require ongoing screening, diagnosis and monitoring for cognitive impairment. Services and policies around these vary widely between settings, and reports from many countries highlight persistent under-diagnosis of cognitive impairment in the months and years after stroke. Missed and delayed diagnosis of post-stroke cognitive impairment, including dementia, are important factors in shaping the experiences of people so affected and their family members, especially in low- and middle-income countries. Drawing upon ethnographic research conducted in Malaysia, this article draws upon three case studies to examine the continued health-seeking behaviour after the appearance of salient cognitive and behavioural symptoms that occurred after stroke. Findings highlight the challenges in getting formal diagnostic clarity for cognitive and behavioural symptoms in a rural setting within a middle-income country. No study participants sought help for memory or cognitive problems, partly due to limited lay awareness of cognitive impairment but more significantly due to health service factors. Despite their elevated risk for dementia, participants were not monitored for cognitive impairment during any follow-up care in various health facilities. Furthermore, caregivers' attempts to seek help when behavioural issues became untenable were met with multiple health system barriers. The journey was complicated by the meanings attached to the reactions towards cognitive symptoms at the community level. We suggest that strategies seek to increase the awareness of post-stroke cognitive and behavioural symptoms, and incorporate clear treatment pathways into the long-term care plans of community-dwelling stroke survivors.

## Introduction

Cognitive impairment (CI) is a frequent complication in stroke survivors (1) and predicts post-stroke death, dependency, and institutionalisation (2–4). Cognitive status offers a way to understand the extent of cognitive impairment and is assessed across a continuous spectrum ranging from no cognitive impairment (NCI) to dementia. While cognitive decline may continue post-stroke, up to 20% of patients with cognitive impairment show improvement (5). While most improvements occur in the first 3 months, recovery may continue for at least the first-year post-stroke. However, stroke survivors can develop delayed dementia beyond the initial 3 months. A registry-based study with mean follow up time of 3.79 years found that, in elderly stroke patients aged above 75 years old, 23.7% developed delayed dementia post-stroke (6). Previous research estimated that 10% of patients had dementia prior to their first stroke, 10% developed dementia after first-ever stroke (7), and approximately one-third experienced dementia following recurrent stroke (8). A 10 -year longitudinal study of people with no stroke at baseline showed that individuals who experienced a stroke during the observation period had faster memory decline than those who remained stroke-free throughout (9). Further, the presence of both post-stroke CI (10) and accelerated post-stroke cognitive decline (11) were independently associated with recurrent stroke. Hence, the recognition of CI in this high-risk group is vital for early intervention and improved management.

The importance of diagnosing and monitoring cognitive impairment after stroke has been repeatedly stressed (12–14). However, reports of under-diagnosis of cognitive impairment remain a common theme in stroke. The onus of the detection of cognitive impairment is often placed in the hands of the healthcare practitioner, and recommendations for post-stroke monitoring emphasises cognitive screening during presentations in hospitals or healthcare facilities. But in reality, health practitioners often miss out on cognitive impairment due to the inherent bias towards physical recovery (in the case of stroke) (15). Delays in the presentation of a person suspected to have cognitive impairment or dementia to health facilities may also play a role, and is commonly attributed to various reasons shaped by the cultural and social environment. This includes: a consideration of memory problems as part of normal ageing (16–18), stigma (17), failure of family members or primary caregivers to recognise symptoms (19–21), and familial disagreements on the symptoms and course of action (17, 20, 22). A timely diagnosis of dementia is beneficial in many ways. Affected individuals and their caregivers or family members can be referred to treatment and support services to enable future planning and better management of disease-related symptoms (23, 24).

Dementia in itself is under-recognised and under-diagnosed in low-and-middle-income countries (LMIC) (25). Contextual factors such as stigma (25), different understandings of disease (26–29), and generally low expectations of abilities in older people (30) contribute to lower awareness and recognition of dementia. LMICs also struggle with limited resources within a health system where acute and other chronic conditions create competing priorities. However, as LMICs are projected to be home for the majority of people living with dementia by 2030 (22), there is a need to better understand how contextual factors interact with health system factors in order to plan for programmes and strategies that will address the underlying issues related to disease recognition and diagnosis.

Malaysia is a middle-income country that is experiencing a demographic transition, edging towards population ageing and increasing prevalence of non-communicable diseases among older adults (31). The Malaysian health system is comprised of both public and private sectors. The majority of the healthcare system is supported by the public sector, with a higher concentration of the private sector in urban areas. In urban areas, where most health facilities (in both private and public sectors) are centred (32), shortages are evident due to the private sector focused on curative services as opposed to long-term chronic care (33). In rural areas, the majority of patients with chronic medical conditions receive their care from the public sector but with less than optimal disease control (34–38). Stroke rehabilitation remains a challenge in Malaysia, where there are there is a shortage in rehabilitation specialists and units (39), and poorly coordinated and fragmented post-stroke care and rehabilitation (40). There is also no information on the experiences of stroke survivors with CI in the community. In this article, we report the continued health-seeking behaviour of three elderly stroke survivors living in the rural community with salient cognitive and behavioural symptoms that occurred after stroke.

## Materials and Methods

### Participants

Participants were recruited as part of a larger ethnographic study on stroke recovery using the South East Asia Community Observatory (SEACO) research platform. SEACO is a demographic and health surveillance system (DHSS) located in the district of Segamat in Johor, the southernmost state of peninsular Malaysia (41). This article focuses on three case studies, drawn from a sub-study of 18 people with stroke and their caregivers. The three participants (two women and one man) sampled for this analysis were aged between 72 and 80 years old. They were all of Malaysian-Chinese descent, and lived in the same village. All three participants lived with their caregivers.

Participation in the study was voluntary and the study was approved by Monash University Human Research Ethics Committee (CF14/315 – 2014000105). A detailed description of the consent process has been previously described (42).

### Methodology

The detailed methodology for the ethnographic study has been described elsewhere (42, 43).

Stroke survivors were identified during two annual health data collection rounds conducted by SEACO. Those who reported stroke were then visited, in order to assess their suitability and willingness to be enrolled in the study. A community key informant referred other stroke survivors who were not captured in the annual round to the study team.

Ethnography was chosen as a research approach in order to gain an in-depth insider understanding of people's behaviours and practises, and what they meant to the participants. Ethnographic research draws upon fieldnotes, observations, interviews and even objects, which are triangulated to produce comprehensive and in-depth understandings of the social environment. Through participant observation, researchers can obtain insights into social practises and behaviour that are not readily apparent to the public eye. Compared with some other forms of qualitative research, ethnographic research can be more difficult to undertake due to this complexity. The need to spend relatively long periods of time gathering data from different sources, with the goal of producing a detailed account of a particular setting, means it is resource- and time-intensive. Because of this intensive approach, sample sizes are typically smaller. This is reflected in the current study, where the first author (KHY) worked with a small number of individuals in a defined geographic area.

The study was located within a single village, and sought to examine the experiences of Malaysian-Chinese stroke survivors and their health seeking pathways, with a special focus on cognitive decline after stroke. The main study on stroke and CI had 18 participants (42, 43). Three community-dwelling participants with salient cognitive and behavioural symptoms after stroke were sampled from this larger ethnographic study. These participants were selected because a) they had a continuous regular engagement with healthcare facilities and were considered compliant to follow-ups, b) they had responsive caregivers who were willing to participate in the study as informants, and c) they all had salient cognitive and behavioural symptoms observed and reported in detail by their caregivers. Within these cases, we examined the missed opportunities for CI diagnosis.

The case narratives of participants were constructed from in-depth interviews, observations and extensive fieldnotes collected over the span of 1 year (July 2015 to June 2016). Several scales were also administered and rated at the beginning of the study in order to capture the current functional state of participants: *Modified Rankin Scale (mRS)* (44), *Patient Health Questionnaire, 9 items(PHQ-9)* (45)*, Montreal Cognitive Assessment (MoCa) basic* (46) and the EuroQol-5D (*EQ5D)* (47) *Visual Analogue Scale (VAS)*.

As this study was part of a larger study on stroke, the initial interview typically started with questions on stroke experience, for example, Why did you think stroke happened? How do you think this should be treated? Who did you call to ask for help? What did you think happened? Questions exploring memory concerns were asked subsequently, including How is your memory? Depending on participants' responses, these questions were then followed up with probing questions. These included questions such as: Do you think you have memory problems (or concerns)? Why did you think your memory worsened? Findings on understandings of stroke and CI were reported elsewhere (42, 43).

Subsequently, the researcher explored the choices made by participants and discussed perceptions of stroke, memory (or aspects of cognition) and health. Contextual issues that may have shaped participants' interaction with health services, including uptake, and impact of their actions in health-seeking were also explored.The first author [KHY] lived in the participants' village during the data collection phase, observing how participants lived and how they carried out daily routines. KHY also accompanied participants and their family members during some of their visits to health facilities, and documented her observations though fieldnotes. All participant names in this article are pseudonyms.

## Findings

### Case 1—Mr Pi

At the time of the study, Mr Pi (72 years old) was 4 years post-stroke and living with his adult daughter (Meijie) and wife (see Table 1). He was usually in his wheelchair and could stand but was not able to walk. He was dependent on his family for all aspects of his life, from toileting and transfers to meal preparation, although he could eat on his own. His diabetic medication was also managed by his caregivers. His scope of activities was therefore limited and not high in complexity. He also did not like to talk much after his stroke. At first glance, it would seem that he was a silently compliant person, living peacefully at home, taken care of by his family. However, it was clear that he had cognitive deficits. This was reflected in his cognitive screening, where he only managed 10 out of the maximum 30 points [*via* the Montreal Cognitive Assessment (48) was administered to him as part of the larger study]. He demonstrated challenging behavioural issues, such as fluctuating moods (resulting in temper tantrums), and occasionally intentionally urinated on the walls of his bedroom, which stressed his family. His behaviour was construed as “naughty” but harmless. Meijie felt that, compared to his previous state where he was completely bedbound and on a feeding tube (for the first month post-stroke), his current condition was a lot better and that he had “stabilised” because he could sit up and could eat well.

**Table 1:** Characteristics of participants in case studies.

**Participant**	**Age**	**Years since stroke**	**Household stucture**	**MoCA score**	**Comorbidities**	**Physical condition at enrolment**
Mr Pi	72	4	Lived with wife and adult children	10	Diabetes	Left-sided weakness, on wheelchair, needed help with transfers.
Madam Lu	80	1	Lived with husband	8	High blood pressure, high cholesterol, arthritis	Left leg weakness and numbness, needed a walking frame to get around
Madam Nu	78	1	Lived with husband, adult children and grandchildren	19	Atrial fibrillation, high blood pressure, diabetes	Slight left facial droop, slight weakness on left hand, ambulated independently.

Meijie and Mrs Pi monitored Mr Pi's health in the local government health clinic within the village. Still, they had never discussed any of his behavioural issues with the healthcare provider as they did not think that the issues were related to the stroke. Mr Pi's consultations with his healthcare providers were generally short (regular checking of vital signs, blood pressure and blood tests reviews), and not conducive for in-depth discussions regarding Mr Pi's management at home. Especially for chronic stroke survivors like Mr Pi, it was assumed that the carer could manage and that, if they had any issues, caregivers themselves would bring them up with the doctor. On the other hand, Meijie had no expectations that anyone in the health clinic would be able to help her, nor was she able to explicitly verbalise what aspect she wanted help with. Even when explicitly asked if there she desired any help in the care of her father, Meijie responded, “Can't think of anything. [I] just take care of him like this.”

#### Analysis

Meijie's perception of Mr Pi's health was influenced by his previous (poor health) state, and her expectation of a “healthier” body was based on this lower baseline. Because he had put on weight, could sit up on his own, and communicate his likes and dislikes to a certain degree, he was considered “okay.” His current state of abilities and behavioural issues were normalised as part of who he was. His family had adjusted to his current state without any expectation of further improvements. This normalisation is similar to findings from India, where the high tolerance of impairments and low expectations for older people was borne out in the low reporting rates or health-seeking for memory issues by their family members (30). Despite regular follow-ups as part of his health management plan, Mr Pi's behavioural and cognitive issues were not detected by his health providers, because his caregivers did not feel that these issues were significant. There was also a lack of monitoring of his cognitive status. Mr Pi was never administered any formal assessments for his cognitive status throughout his care.

### Case 2—Madam Lu

At the time of the study, Madam Lu (80 years old) was 1 year post-stroke, and living with her husband, Mr Xing (79 years old) (see Table 1). She still had remaining leg weakness and needed to use the walking frame for stability. She had high blood pressure and high cholesterol and was put on medications. Explicit cognitive symptoms were observed in Madam Lu. Her husband managed her medications for her, serving the appropriate doses to her as she would otherwise forget to take them or muddle up the dosage. Both she and her husband were aware of her memory decline, but this was normalised even though she depended on Mr Xing for some, but not all, of her activities of daily living. He did the majority of the household chores. While she used to handle all household chores on her own, by the time of the study, Madam Lu was only able to manage simple tasks in food preparation (plucking vegetables) or folding small items of clothing. Her cognitive issues became more apparent when they shifted into a newly renovated home a few streets away; Madam Lu sometimes became disoriented and asked to be brought to back to her “real home” because she forgot that she had moved. Mr Xing became increasingly puzzled with her behaviour, which included moments when she would ask to die, which he interpreted as evidence that she might be having some mental health issues.

Throughout this time, Madam Lu regularly attended the government health clinic for blood pressure and cholesterol medications but, like Meijie (the caregiver from Case 1), Mr Xing did not bring up these issues to the healthcare provider. Instead, he asked his son to take Madam Lu to the private hospital to consult a psychiatric specialist. Madam Lu, on the other hand, was still preoccupied with her leg weakness. She complained about her leg when her son came to take her to the private hospital; she was brought to the orthopaedic specialist to consult on her leg instead of the psychiatrist. Mr Xing did not want to make a fuss because he felt that the decision-making regarding the choice of health provider lay with his son, who paid for Madam Lu's medical expenses at the private hospital.

This decision to remain silent was borne out negatively over time. One and a half years after her stroke, Madam Lu lost her balance at home and experienced a bad fall, after which she became bedbound. Mr Xing felt that it was because she had forgotten to hold on to her walking frame when making her way to the bathroom, and had therefore lost her balance and fallen. After the incident, Mr Xing simply could not cope alone at home with her, so she was shifted to her son's home to be cared for by him and his family. At that point, her medical care was transferred to the nearest public hospital located in the same town and, from then on, her mental state worsened:

She just sits there and lies down there you know. She is not very spirited you know. she does not really want to talk. The first time when I went to see her, she was sitting there and her eyes were wide open. When people asked her who came to see her, she could still tell [who visited]… The second time when I went to see her. she lay there with her eyes closed. (Mr Xing)

Before she was confined to bed, she was socially active and generally chatty. After her fall, she became very depressed and quickly deteriorated physically and mentally. Mr Xing felt helpless as he watched the deterioration of her mental state, but it was not something that he thought needed management at that time. Madam Lu passed away in her sleep 2 months after she moved to her son's home.

#### Analysis

Different levels of recognition of the impact of CI meant that participants received varying levels of support to manage their well-being. Earlier recognition and diagnosis of CI and dementia in clinical settings has been advocated because it provided patients and caregivers with earlier support and training to cope at home (38, 39). Mr Xing perceived that there was a need to address her issues and had sought out resources from their son, an external resident. However, due to his lack of understanding of Madam Lu's condition, their son took her to an orthopaedic specialist instead of a professional who could address her psychological and behavioural issues. In the end, because her son provided the resources for her specialist care, Madam Lu was not able to access services relevant to the needs perceived by Mr Xing. Even though she had been in contact with various healthcare providers, her cognitive issues were not detected in any of the health facilities in either public or private sectors. Again, this points to the lack of monitoring of patients at-risk for dementia by health service providers. Arguably, Mr Xing and Madam Lu's confusion, distress, and helplessness may have been alleviated to a certain degree if the impact of CI was recognised and supported by healthcare providers.

### Case 3—Madam Nu

At the time of the study Madam Nu (78 years old) was 1 year post-stroke and lived with her husband, children and grandchildren (see Table 1). She had recovered well after stroke and was only left with facial numbness. Her daughter, Huajie, was in charge of the arrangements and transport for Madam Nu's health visits, including regular follow-up visits to the hospital in another town for a heart-related condition. Huajie had noticed that Madam Nu tended to dwell on past memories, and did not always remember what happened more recently. While Madam Nu was aware that her memory had declined, she was unperturbed as the deficits did not interfere with her daily activities. Household chores were done by the younger generation and she usually spent her time watching television.

Huajie became very concerned when Madam Nu's behaviour started to disrupt home life. Madam Nu experienced increasingly frequent periods of poor temper, which created uncomfortable situations for her family members. She would often jump to misconstrued conclusions based on incomplete information, as she had forgotten some of the events that happened in between. Madam Nu's behaviour also caused embarrassment for her family, as some of the storeys she told to her neighbours reflected badly on her family members. Her memory decline not only meant she forgot the small everyday acts of care performed by her family, but also amplified her resistance towards their help or suggestions for activities. Incidents that happened at home were told to other people from a negative angle, with omissions or exaggerations of some details contrary to what really happened. This then strained Madam Nu's relationships with her family members as she created conflicts out of normal everyday interactions.

As the problems with Madam Nu escalated, Huajie tried to suggest a visit to see the psychiatrist, but this was met with intense objection from Madam Nu as she not feel that there was anything wrong with her. She also did not realise that her memory deficits caused some of the misunderstandings that resulted in her suspicion and anger. Huajie then attempted to bring up her mother's behavioural issues with their regular health practitioner at during a follow-up consultation:

I told [the doctor] that my mother's temper is getting worse. [She] keeps scolding people, [so could they recommend] any method or any medication to control her [behaviour]? The doctor just smiled at me. I am not sure if he thought that we were unable to communicate with my mother or that we complain that our mother is not good or because of our socioeconomic status. He did not further ask properly.

Huajie had hoped to get a referral or some suggestions from the doctor to help manage Madam Nu's mood and occasionally erratic behaviour. Her concerns were brushed aside by the attending doctor. Huajie was frustrated with the doctor's reaction, which.she felt reflected an incorrect comprehension of the extent to which she and her family had gone to try and understand Madam Nu.

#### Analysis

Family members were not always guaranteed any response or helpful advice even when they did report behavioural issues to health providers. Huajie, who finally decided to bring up the matter with Madam Nu's regular healthcare provider after her behavioural symptoms escalated, was dismissed by the doctor. This left Huajie at a loss—she had perceived a need to address such issues, but received no support to do so. In this case, the healthcare provider did not think that the patient needed to be assessed for further management, which resulted in Huajie not having access to appropriate care which was crucial for addressing the Madam Nu's behavioural issues in the home setting.

## Discussion

Individuals in this article did not seek help for memory or cognitive problems. Instead, behavioural issues prompted caregivers to seek help. This phenomenon is not surprising and reflected in the same trends found in more well-resourced settings (49). In the current context, health service factors contributed significantly to this phenomenon.

Even though stroke survivors are known to have elevated risk of progressing to dementia, the three study participants were not monitored for cognitive impairment during any follow-up care in various health facilities. According to the participants and their caregivers, none had undergone any form of formal assessment of their cognitive abilities in any of the health facilities they attended, not even during the acute stage of stroke. This was also the case for all participants in the broader study. Contextual issues shaped how they viewed CI, and the obvious emphasis on physical abilities were barriers to the diagnosis of CI or behavioural issues (42). The lack of pathways for stroke after hospitalisation extended to other symptoms, including CI and other psychosocial issues, which increased the difficulties in getting a proper diagnosis. Study participants and their families simply had no clear idea of what to do when faced with these issues, resulting in delays to seeking help from health service providers or not seeking help at all. Families experienced some degree of distress when dealing with the changed behaviour of their loved ones and were stuck in uncertainty. They knew that something was wrong, but could not quite place what this was, and so were not sure of how to go about getting help. This points to the need to implement a care plan or pathway for stroke survivors in which cognitive assessments are routinely undertaken, as per recommendations in international guidelines (50, 51).

The health providers consulted in these three cases did not seem to be picking up distress signals from the family caregiver when they reported mood and behavioural changes, regardless of the time since stroke, the severity of the CI symptoms, or even reports of mood and behavioural issues. None of these were considered by healthcare providers who were in contact with stroke survivors in the community. In Malaysian settings, behavioural and psychological symptoms significantly contribute to caregiver burden more than the actual cognitive impairment in patients with dementia (52). Therefore, there is a need to strengthen the awareness, knowledge and attitudes of stroke survivors and their caregivers regarding cognitive and behavioural signs, symptoms and changes after stroke—all factors that need attention and reporting. Caregivers should also be educated on the resources and facilities that they can contact when they have doubts about the symptoms or progression of their loved ones. In doing so, caregivers will be able to actively recognise symptoms and make the first step for discussion with healthcare providers, which can lead to suitable treatment or further referrals. The strategies to strengthen awareness need to consider family members' and patients' lay understandings of their condition and the interactions within the households' family structure.

In this paper, we have highlighted the challenges in getting formal diagnostic clarity for cognitive and behavioural symptoms in a rural setting within a middle-income country. We suggest that strategies seek to increase the awareness of post-stroke cognitive and behavioural symptoms, and incorporate clear treatment pathways into the long-term care plans of community-dwelling stroke survivors.

The emphasis of the original study from which the case narratives were extracted from is from the perspective of the stroke survivors and their family units, and focuses less on the perspective of healthcare services providers. One of the reasons for the lack of inclusion of the perspective from healthcare providers is the need for additional layers of ethical and institutional approvals in obtaining formal interviews (especially concerning Ministry of Health employees and facilities). Future studies should include healthcare providers' perspectives in terms of long-term follow up and actual management in the community.

## Data Availability Statement

The datasets presented in this article are not readily available because we are unable to publicly share the data due to restrictions related to ethical approval. We can share other publications and original theses arising from this project. Requests to access the datasets should be directed to NW, narelle.warren@monash.edu.

## Ethics Statement

The studies involving human participants were reviewed and approved by Monash University Human Research Ethics Committee. The patients/participants provided their written informed consent to participate in this study. Written informed consent was obtained from the individual(s) for the publication of any potentially identifiable images or data included in this article.

## Author Contributions

KY designed the cognitive screening component of the study, and collected and analysed all data. NW, PA, and DR designed the study, obtained funding and ethical approval, and guided the data analysis. All authors contributed to participant recruitment and writing the manuscript.

## Conflict of Interest

The authors declare that the research was conducted in the absence of any commercial or financial relationships that could be construed as a potential conflict of interest.
